# Supplementary material for: Increased Biomass and Polyhydroxybutyrate Production by Synechocystis sp. PCC 6803 Overexpressing RuBisCO Genes
Source: Int J Mol Sci. 2023 Mar 29;24(7):6415. doi: 10.3390/ijms24076415 (PMC10094337; doi:10.3390/ijms24076415)

Supplementary Information

Table S1 Primers used in this study

Target gene	Name	Sequence 5' to 3'	Purpose of primers	Product size (bp)	T_m(°C)/Cycles
rbcL	rbcL_F	GGACTAGTATGGTACAAGCCAAAGC	PCR	1413	58 °C/30
	rbcL_R	AAAACTGCAGTTAGAGGGTATCCATGGC			
rbcX	rbcX_F	GCTCTAGATTACCGGGTAGAGTGTTCAT	PCR / RT-PCR	433	58 °C/26
	rbcX_R	GGACTAGTTTAGGACGGGGGAGAATCGTT			
rbcS	rbcS_F	GGACTAAGTCATCAGCAAGGAAAACTTTTA	PCR / RT-PCR	373	58 °C/26
	rbcS_R	AACTGCACTTAGTAACGGCCTTGGTTTTGG			
Up*psbA2*	UppsbA2_F	TGCCTGTCAGCAAAACAACTT	PCR	-	58 °C
DS*psbA2*	DSpsbA2_R	CGAGGGCAATCATCAATTCCG			
CM	CM_F	GAGTTGATCGGGCACGTAAG	PCR	899	58 °C
	CM_R	CTCGAGGCTTGGATTCTCTCAC			
16S rRNA	16S_F	AGTTCTGACGGTACCTGATGA	RT-PCR	521	59 °C/8
	16S_R	GTCAAGCCTTGGTAAGGTTCT			
RT_*rbcL*	RTrbcL_F	GGTATCACCATGGGCTTCGTTGACCT	RT-PCR	411	58 °C/30
	RTrbcL_R	AAAACTGCAGTTAGAGGGTATCCATGGC			
RT_*phaA*	RTphaA_F	CATGATGGTTTGACGGACAG	RT-PCR	310	58 °C/30
	RTphaA_R	AGACTTTCCACGGTGGTGTC			
RT_*phaB*	RTphaB_F	GCTCCATTGTGGCCATTAGT	RT-PCR	255	58 °C/26
	RTphaB_R	CAATTTCCTCCGGTTTACCA			
RT_*phaC*	RTphaC_F	GGGCACATTTAGCCTGTGTT	RT-PCR	267	58 °C/26
	RTphaC_R	CCCATAATATCGGGCACATC			
RT_*phaE*	RTphaE_F	GGCCATGGCAGACTATCAAT	RT-PCR	384	58 °C/23
	RTphaE_R	TAGCCTGGGTTTGCTTCTGT			
RT_*plsX*	RTplsX_F	AAGGGGTGGTGGAAATGGAA	RT-PCR	467	59 °C/28
	RTplsX_R	AAGTACGTCCCTTCCTTCGG			
RT_*plsC*	RTplsC_F	TCTCTACCGGGGCTTGAAATG	RT-PCR	508	58 °C/32
	RTplsC_R	CGCCTTACCAATGCGAATAGT			
RT_*glgX*	RTglgX_F	GAGCTTCATCGAGGACGGAA	RT-PCR	360	58 °C/26
	RTglgX_R	GCCCGAATTGGGGTTGCGGG			

Table S2 The band intensity data detected by Syngene® Gel Documentation (Syngene, Frederick, MD, USA) in Figures 8 and 9.

(A) The band intensity of each gene (transcript level)

Genes	Normal BG_{11}			BG_{11}-N			BG_{11}-P			BG_{11}-NP		
	WTc	OX *rbcL*	OX *rbcLXS*	WTc	OX *rbcL*	OX *rbcLXS*	WTc	OX *rbcL*	OX *rbcLXS*	WTc	OX *rbcL*	OX *rbcLXS*
phaA	0.1	0.7	0.7	5.0	6.2	5.5	1.0	4.0	5.0	2.0	4.0	5.5
phaB	0.3	0.3	0.3	3.0	3.5	3.5	0.5	0.5	0.5	1.2	4.0	3.8
phaC	0.2	0.2	0.1	2.5	4.0	3.2	0.8	0.9	1.0	1.0	3.0	3.0
phaE	0.1	0.2	0.2	0.3	0.3	0.3	0.3	0.4	0.4	0.1	0.1	0.1
plsX	0.2	0.2	0.1	4.0	4.0	0.8	0.4	0.8	1.8	0.4	0.4	0.4
plsC	1.2	0.8	2.0	2.5	3.0	2.0	3.1	3.4	3.6	1.2	1.3	1.6
glgX	0.2	0.2	0.2	0.6	2.7	2.5	1.0	1.5	1.6	1.2	2.0	2.5
16S	1.0	1.0	1.0	1.0	1.0	1.0	1.0	1.0	1.0	1.0	0.8	0.8

(B) The ratios of band intensity of each gene/*16s*

Genes	Normal BG_{11}			BG_{11}-N			BG_{11}-P			BG_{11}-NP		
	WTc	OX *rbcL*	OX *rbcLXS*	WTc	OX *rbcL*	OX *rbcLXS*	WTc	OX *rbcL*	OX *rbcLXS*	WTc	OX *rbcL*	OX *rbcLXS*
phaA	0.1	0.7	0.7	5.0	6.2	5.5	1.0	4.0	5.0	2.0	5.0	6.9
phaB	0.3	0.3	0.3	3.0	3.5	3.5	0.5	0.5	0.5	1.2	5.0	4.8
phaC	0.2	0.2	0.1	2.5	4.0	3.2	0.8	0.9	1.0	1.0	2.8	2.9
phaE	0.1	0.2	0.2	0.3	0.3	0.3	0.3	0.4	0.4	0.1	0.1	0.1
plsX	0.2	0.2	0.1	4.0	4.0	0.8	0.4	0.8	1.8	0.4	0.5	0.5
plsC	1.2	0.8	2.0	2.5	3.0	2.0	3.1	3.4	3.6	1.2	1.6	2.0
glgX	0.2	0.2	0.2	0.6	2.7	2.5	1.0	1.5	1.6	1.2	2.5	3.1

(C) Fold change value of "band intensity ratio of each gene/*16s* under stress" divided by "band intensity ratio of each gene/*16s* under normal condition", shown in Figure 9.

Genes	BG_{11}-N			BG_{11}-P			BG_{11}-NP		
	WTc	OX *rbcL*	OX *rbcLXS*	WTc	OX *rbcL*	OX *rbcLXS*	WTc	OX *rbcL*	OX *rbcLXS*
phaA	50.0	8.9	7.9	10.0	5.7	7.1	30.0	7.1	9.8
phaB	9.7	10.9	12.5	1.6	1.6	1.8	3.9	15.6	17.0
phaC	12.5	20.0	32.0	4.0	4.5	10.0	5.0	14.0	29.0
phaE	3.0	1.5	1.5	3.0	2.0	2.0	1.0	0.6	0.6
plsX	20.0	20.0	8.0	2.0	4.0	18.0	2.0	2.5	5.0
plsC	2.1	3.8	1.0	2.6	4.3	1.8	1.0	2.0	1.0
glgX	3.0	13.5	12.5	5.0	7.5	8.0	6.0	12.5	15.6

Figure S1 : Agarose gel electrophoresis of RT-PCR product of *rbcL* transcript in *Synechocystis sp.* PCC 6803 grown under normal BG_{11} condition, shown in **Figure 2D**. The *16s* rRNA transcript was used as the reference.

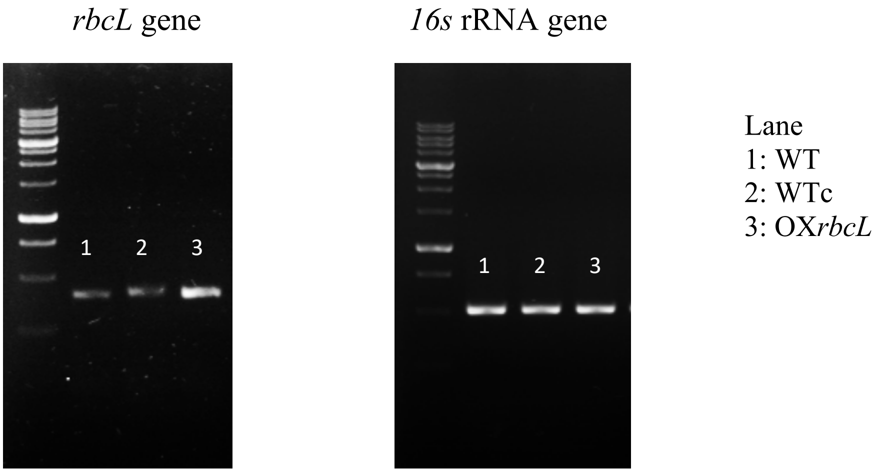

Figure S2 : Agarose gel electrophoresis of RT-PCR product of *rbcL, rbcX, rbcS* transcripts in *Synechocystis sp.* PCC 6803 grown under normal BG_{11} condition, shown in **Figure 2D**. The *16s* rRNA transcript was used as the reference.

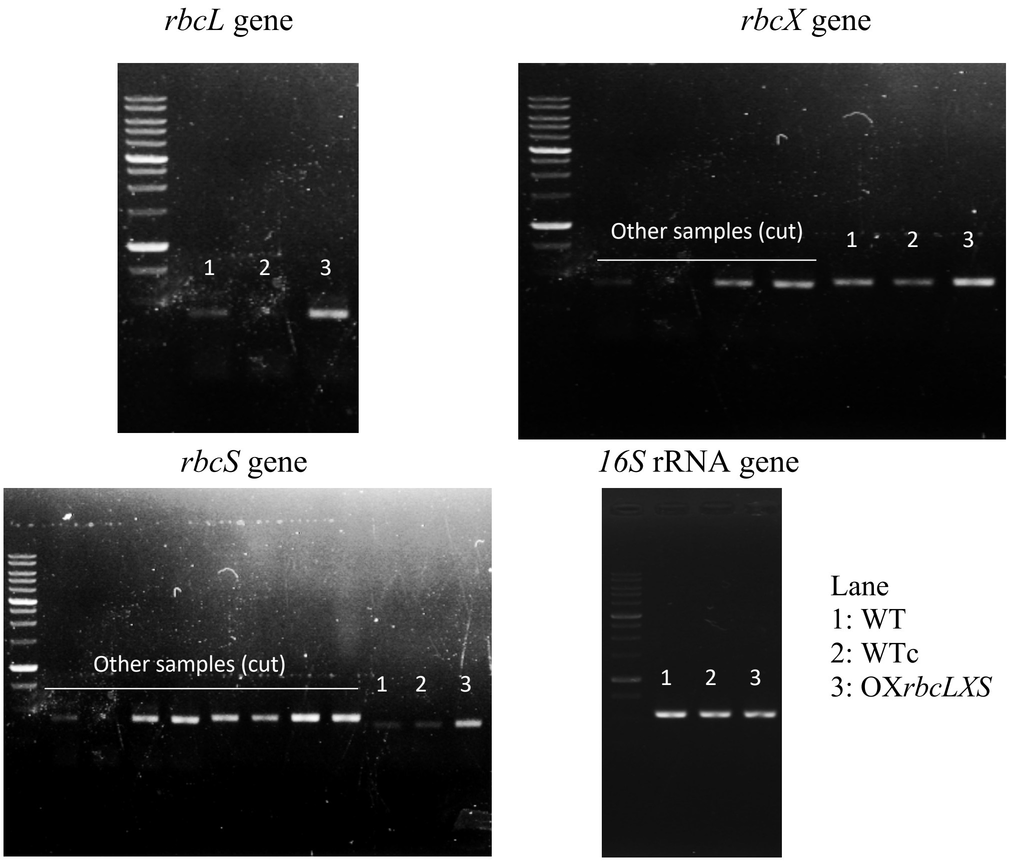

Figure S3 : Agarose gel electrophoresis of RT-PCR product of *phaA, phaB, phaC, phaE, plsX, plsC, rbcS, glgX,* and *16s* transcripts in *Synechocystis sp.* PCC 6803 grown under stresses and normal BG_{11} condition, shown in **Figure 8**. The *16s* rRNA transcript was used as the reference.

phaA (BG_{11})

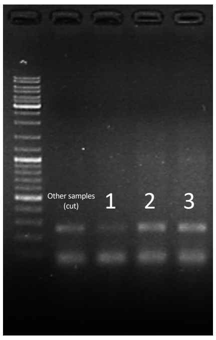

phaA (BG_{11}-N)

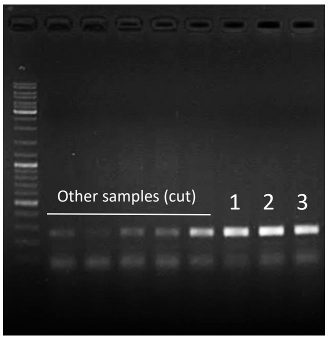

Lane
1: WTc
2: OX*rbcL*
3: OXrbc*LXS*

phaA (BG_{11}-P)

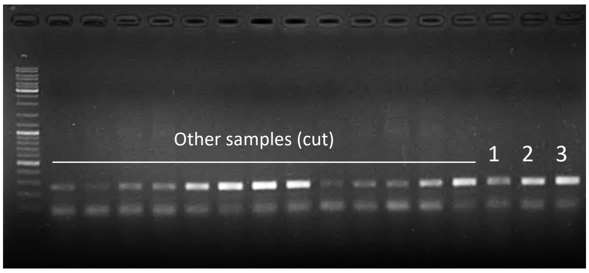

phaA (BG_{11}-NP)

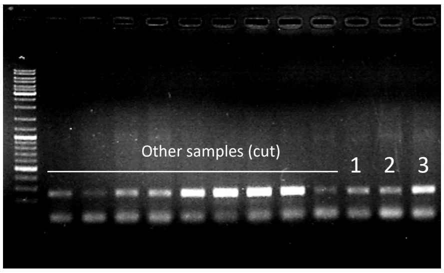

phaB (BG_{11})

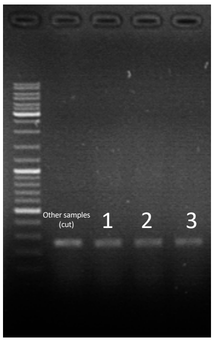

phaB (BG_{11}-N)

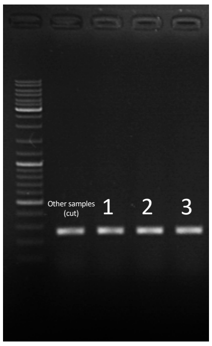

Lane
1: WTc
2: OX*rbcL*
3: OXrbc*LXS*

phaB (BG_{11}-P)

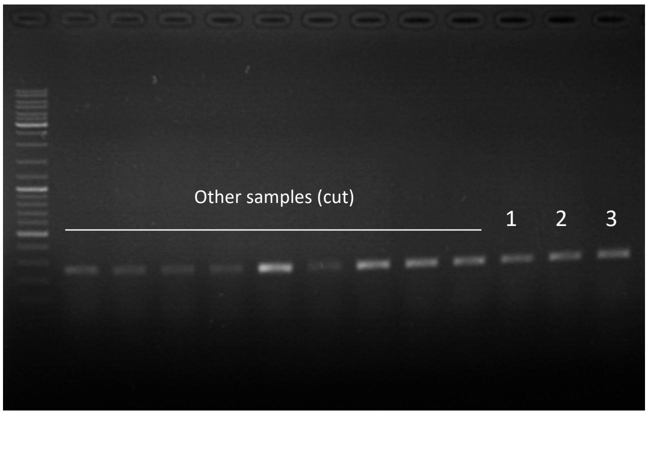

phaB (BG_{11}-NP)

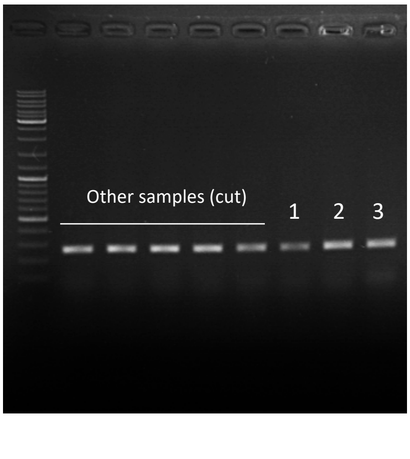

phaC (BG$_{11}$)

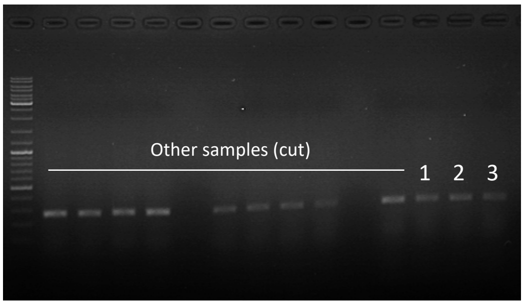

phaC (BG$_{11}$-N)

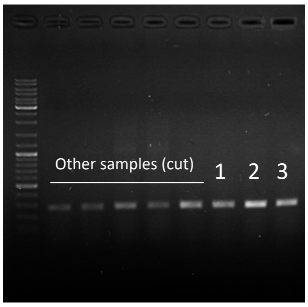

Lane
1: WTc
2: OX*rbcL*
3: OXrbc*LXS*

phaC (BG$_{11}$-P)

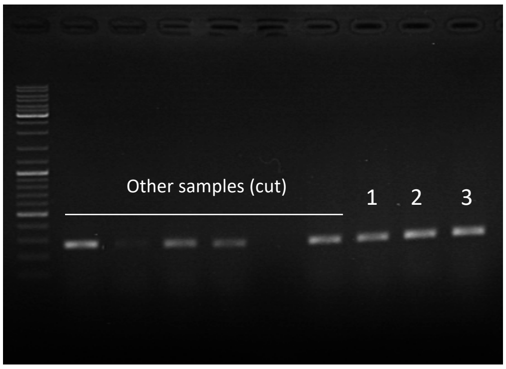

phaC (BG$_{11}$-NP)

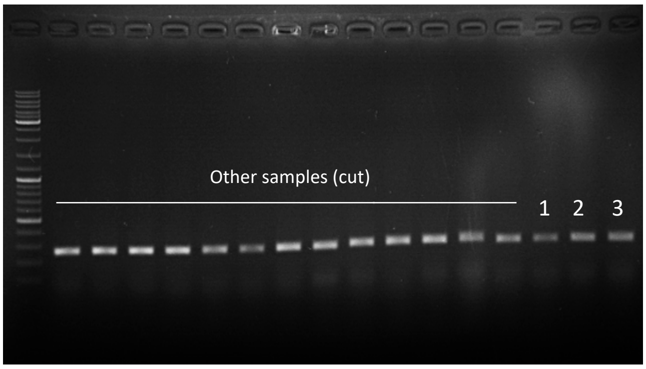

phaE (BG$_{11}$)

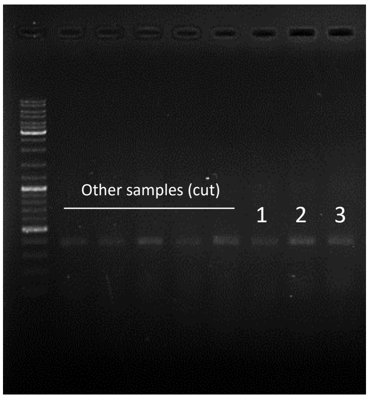

Lane
1: WTc
2: OX*rbcL*
3: OXrbc*LXS*

phaE (BG$_{11}$-N)

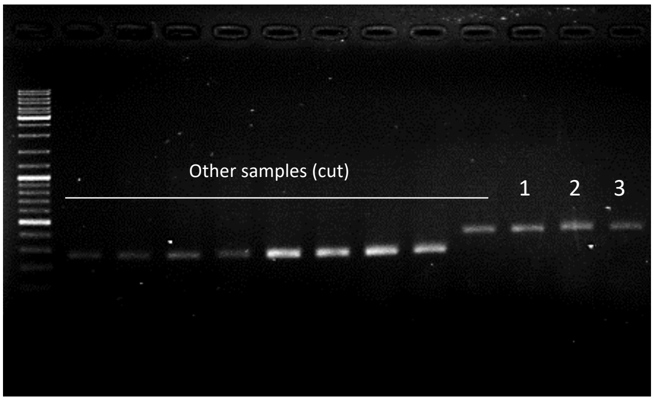

phaE (BG$_{11}$-P)

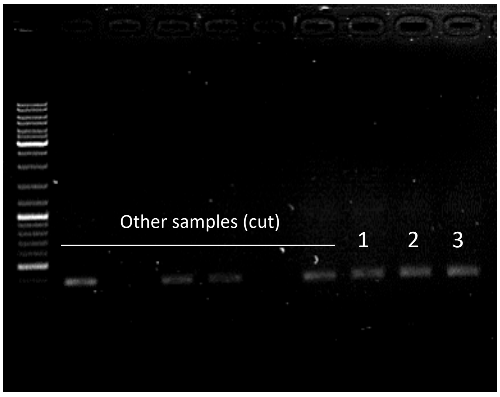

phaE (BG$_{11}$-NP)

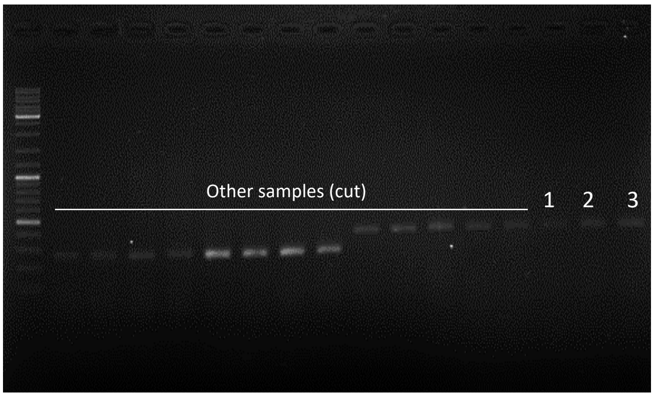

plsX (BG_{11})

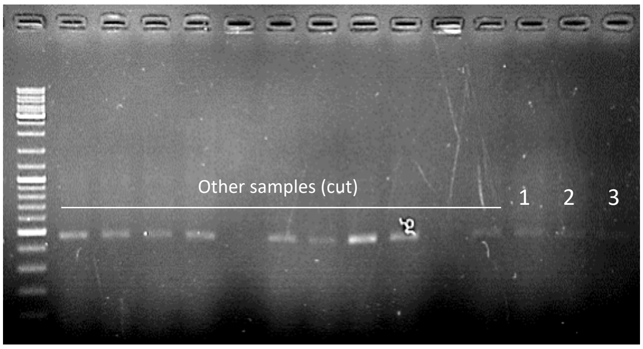

plsX (BG_{11}-N)

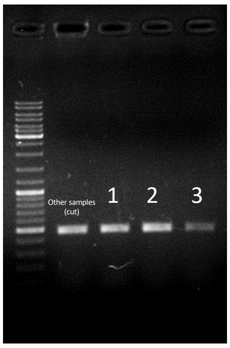

Lane
1: WTc
2: OX*rbcL*
3: OXrbc*LXS*

plsX (BG_{11}-P)

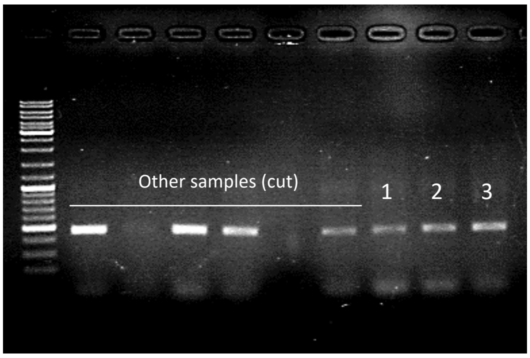

plsX (BG_{11}-NP)

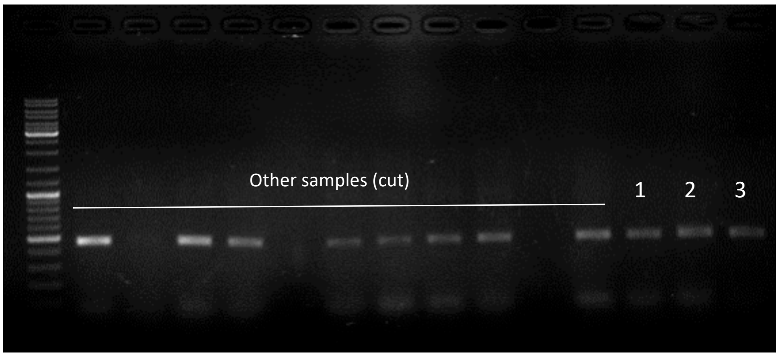

plsC (BG_{11})

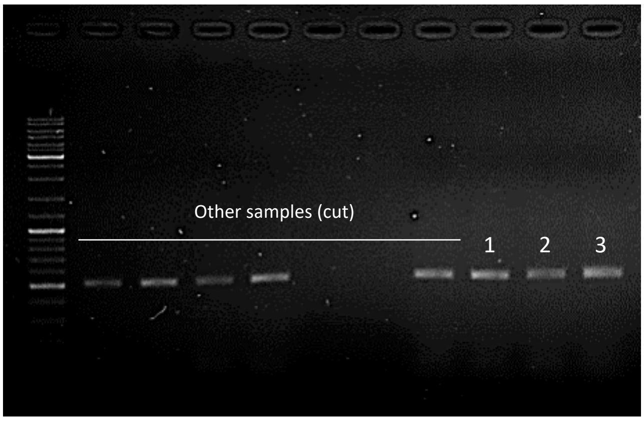

plsC (BG_{11}-N)

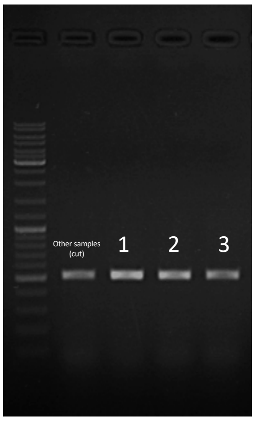

Lane
1: WTc
2: OX*rbcL*
3: OXrbc*LXS*

plsC (BG_{11}-P)

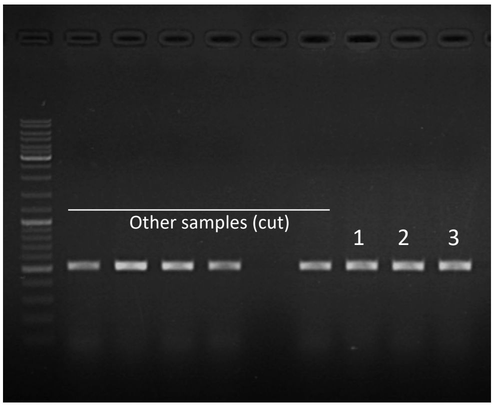

plsC (BG_{11}-NP)

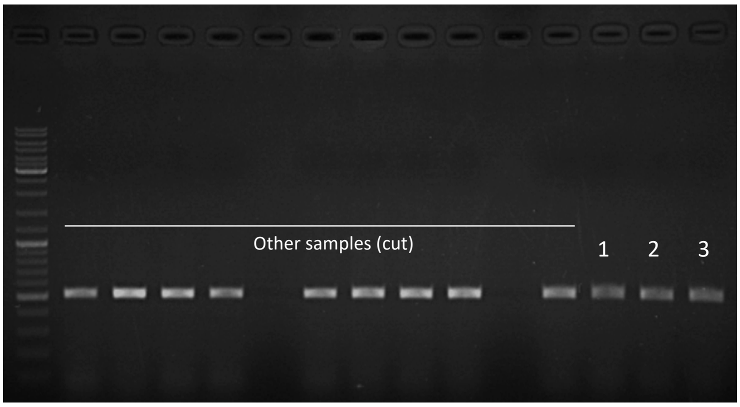

glgX (BG_{11})

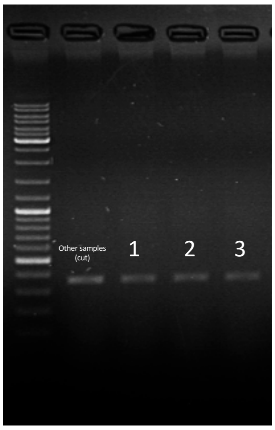

glgX (BG_{11}-N)

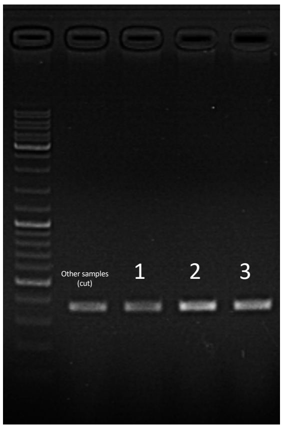

Lane
1: WTc
2: OX*rbcL*
3: OXrbc*LXS*

glgX (BG_{11}-P)

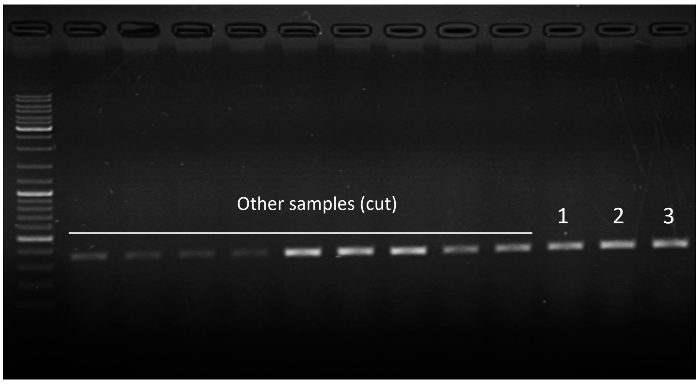

glgX (BG_{11}-NP)

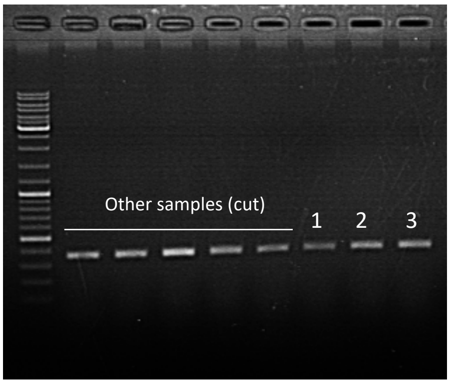

16S rRNA (BG_{11})

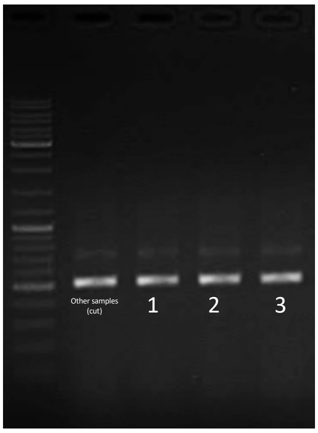

16S rRNA (BG_{11}-N)

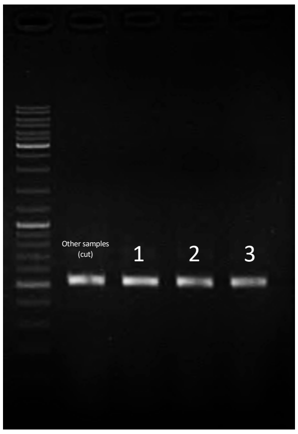

Lane
1: WTc
2: OX*rbcL*
3: OXrbc*LXS*

16S rRNA (BG_{11}-P)

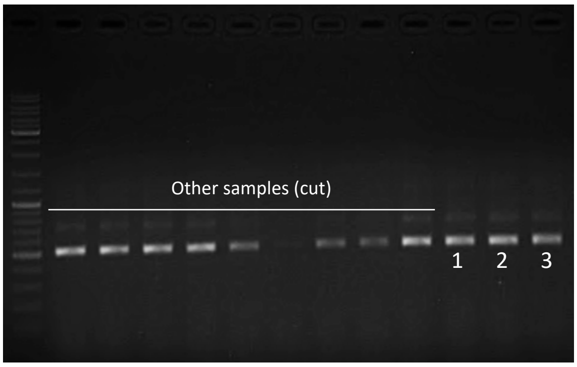

16S rRNA (BG_{11}-NP)

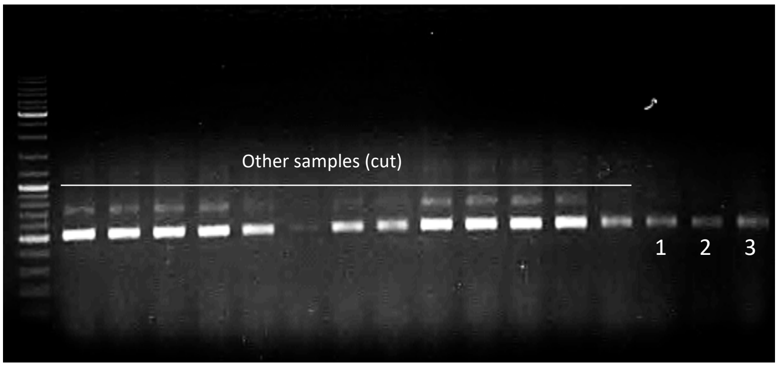

Supplement: Supplementary file 1 [file ijms-24-06415-s001.zip › ijms-2292806-supplementary.pdf]
